# Neck Epithelioid Sarcoma at an Unusual Location Mimicking Lymph Node Metastases of Nasopharyngeal Carcinoma: A Case Report

**DOI:** 10.7759/cureus.56484

**Published:** 2024-03-19

**Authors:** Soufia El Ouardani, Hind Chibani, Fatima Rezzoug, Ayoub Kharkhach, Ouissam Al Jarroudi, Sami Aziz Brahmi, Said Afqir

**Affiliations:** 1 Medical Oncology, Mohammed VI University Hospital, Oujda, MAR; 2 Medical Oncology, Faculty of Medicine and Pharmacy, Mohammed First University, Oujda, MAR; 3 Surgical Oncology, Faculty of Medicine and Pharmacy, Mohammed First University, Oujda, MAR; 4 Surgical Oncology, Mohammed VI University Hospital, Oujda, MAR

**Keywords:** immunohistochemistry staining, chemoresistant, tazemetostat, undifferentiated nasopharyngeal carcinoma, epithelioid sarcoma

## Abstract

Epithelioid sarcoma (ES) is an uncommon soft tissue sarcoma. It is usually located in the extremities and exceptionally in the neck. Its diagnosis constitutes a real challenge which is based on histology and immunohistochemistry staining that must be interpreted with caution given the anatomopathological similarities to other tumors. In this article, we report a case of a 37-year-old man admitted for a locally advanced ES of the neck. There were suspicions of lymph node metastases of nasopharyngeal carcinoma at the first pathological examination. The patient received palliative chemotherapy and was referred to the supportive care department. Through this case, we will discuss the clinical and anatomopathological characteristics and therapeutic options of this extremely rare tumor which poses a diagnostic challenge.

## Introduction

Soft tissue sarcomas are entities of particular and heterogeneous malignancies known for their aggressiveness and rarity [1]. Epithelioid sarcoma (ES) is one of this group that constitutes less than one percent of all soft tissue sarcomas [2]. ES is mostly located in the extremities and preferentially in the hands [2,3]. Cervical localization is extremely rare [4]. The diagnosis is based on pathological and immunohistochemical examination to exclude differential diagnoses [5]. This tumor is highly aggressive and responds poorly to standard anti-cancer treatments [6]. Like all localized soft tissue sarcomas, surgical resection with an R0 margin is the gold standard treatment [7]. Adjuvant radiotherapy can be indicated depending on prognostic factors [7]. There is no standard treatment for metastatic disease; a few molecules have demonstrated clinical benefits with low survival rates [1].

## Case presentation

A 37-year-old male patient was admitted to the oncology center for the treatment of a right-sided cervical mass that appeared and had been gradually increasing in size for four months before admission. The patient did not have any significant medical history. The symptoms worsened with the onset of cervicobrachial neuralgia.

**Figure 1:**
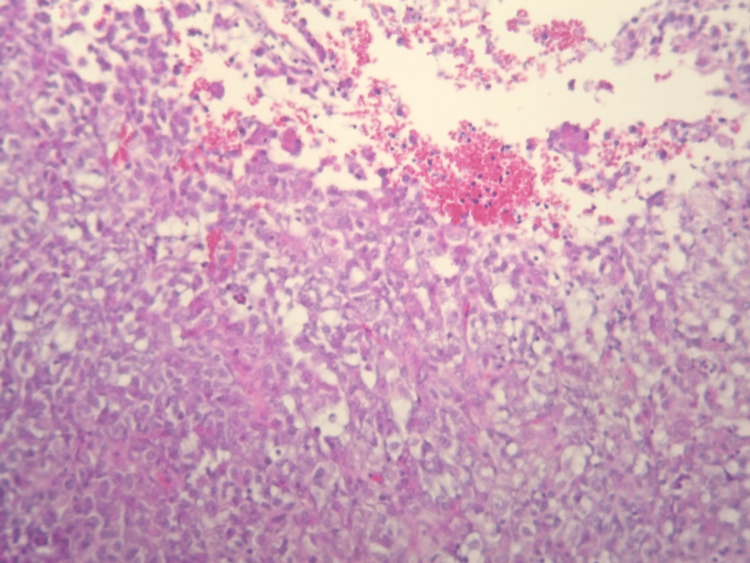
Photomicrograph of the tumor. A malignant proliferation arranged in a sheet, composed of large, non-uniform, and moderately atypical cells. The nucleus has an elongated, ovoid shape, vesicular chromatin, and prominent nucleoli. The cytoplasm is abundant, eosinophilic, and focally vacuolated (H&E, ×100).

**Figure 2:**
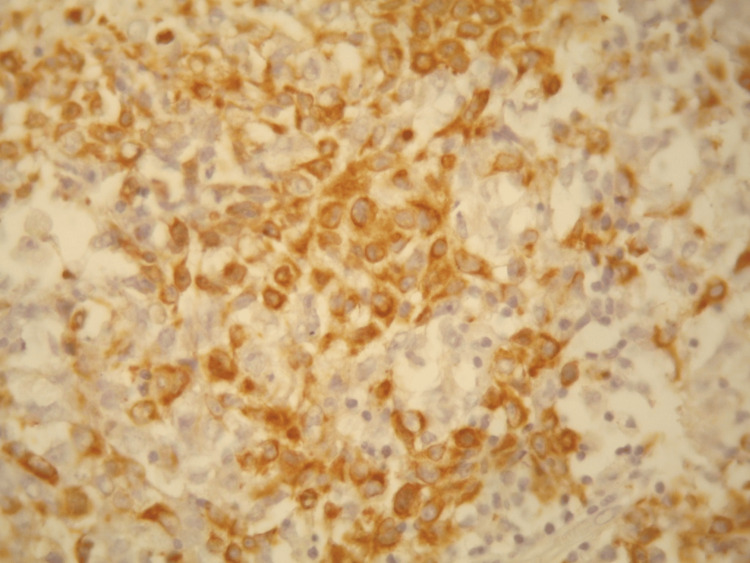
Positive staining of tumor cells by antiAE1-AE2 (DAB, x400).

**Figure 3:**
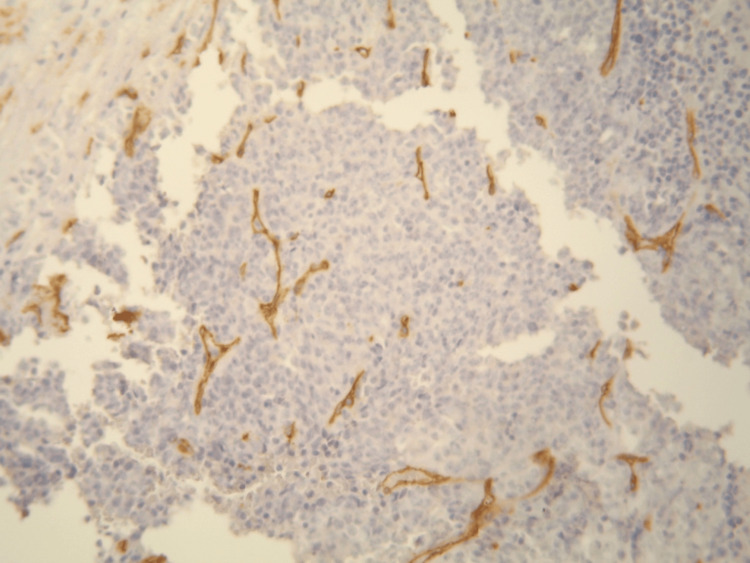
Absence of staining with anti-CD34 antibody (DAB, x200).

**Figure 4:**
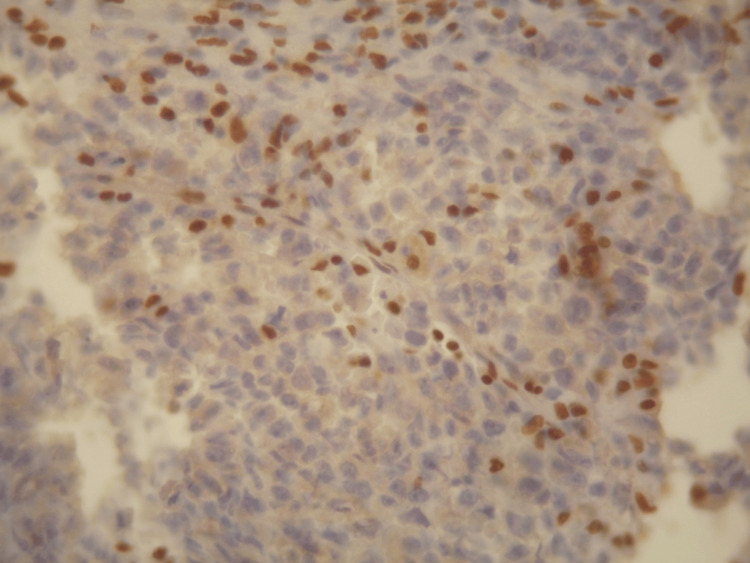
Loss of expression of INI1 in the tumor cells (DAB, x400).

The patient had a good performance status (PS 1). The cervical examination found a huge, firm, immobile mass located on the right side of the neck extended to the clavicular region. The computed tomography (CT) scan of the neck identified a right cervical lymphadenopathy mass, which appeared heterogeneous with varying composition including necrotic areas, causing a mass effect on the right carotid sheath. The surgical biopsy of the mass was in favor of a lymph node location of an undifferentiated malignant tumor arranged in a sheet, with large and moderately atypical cells, leading to suspicion of an undifferentiated carcinoma probably of the nasopharyngeal type (Figure 1). The immunostaining revealed positive results for anti-cytokeratin AE1/AE2, anti-EMA (epithelial membrane antigen), and partially positive for anti-S-100. CD34 was negative in the tumor cells with loss of INI1 (Figures 2-4).

**Figure 5:**
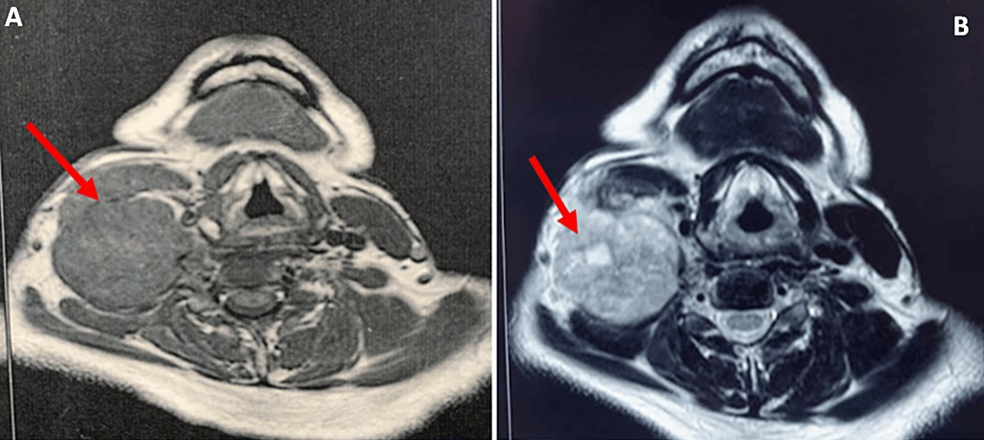
Axial T1-weighted (A) and T2-weighted (B) MRI of the nasopharynx. A cervical nodular mass in the right region (red arrows) that includes areas of necrosis and extensive invasion of the surrounding fat.

Magnetic resonance imaging (MRI) of the nasopharynx showed a nodular mass on the right cervical and clavicular region with areas of necrosis and extensive invasion of the surrounding fat. The presence of bilateral cervical lymphadenopathy with suspicious characteristics and the lack of any anatomic lesion of the nasopharynx had been identified (Figure 5). The patient had no distant metastasis on the thoracic and abdominopelvic CT scan. 

Following a thorough discussion at a multidisciplinary consultation meeting, the diagnosis of cervical ES was made on the basis of the pathological and immunohistochemical profile. The tumor was locally advanced and unresectable and the patient underwent initial chemotherapy using ifosfamide and doxorubicin (AI regimen) with G-CSF support. After three cycles, the patient presented with active bleeding in the cervical mass and he received hemostatic radiotherapy. The evaluation was in favor of pulmonary, bone, and peritoneal metastases with performance status deterioration (PS 4). It was decided to refer him to the palliative care department.

## Discussion

The proximal ES is an unusual and rare tumor, both by its frequency and cervical localization. It constitutes less than one percent of all soft tissue tumors [2]. The first description of the classical distal form was made by Laskowski in 1961 [8] and then declared as a special anatomoclinical form of sarcoma by Enzinger in 1970 [3]. The cervical location remains exceptional and rarely documented in the literature [4,9]. The ES affects young adults, especially men. It typically occurs in the extremities and affects the subcutaneous tissues such as aponeurosis and fascia [5]. This sarcoma is highly aggressive and has a significant potential for metastasis and local recurrence [10].

From a clinical perspective, cervical localization generally translates into an asymptomatic cervical mass. The symptoms such as pain, neuralgia, edema, ulceration, and bleeding appear with tumor progression [7]. This mass can be mistaken sometimes for lymph node metastases of a head and neck malignancy such as nasopharyngeal cancer as was the situation with our patient [4]. Imaging can help to diagnose soft tissue sarcomas, especially MRI is an excellent test to evaluate local extension of soft tissue lesions [11]. The diagnosis of proximal ES is purely pathological, a competent anatomopathologist is required to accurately identify this tumor [12]. The panel of antibodies must be sufficiently extended to ensure a correct diagnosis that matches the immunohistochemical profile [9].

Pathological examination of the proximal form of ES is different from the conventional distal form and it is distinguished by enlarged cells with a prominent cytoplasm organized in nodules that may have a central necrosis [13]. The presence of rhabdoid differentiation in the cells is associated with an unfavorable prognosis [2]. Immunohistochemistry shows positive epithelial markers such as cytokeratin, EMA, and positive conjunctive markers like vimentin and CD34 [10]. It is worth noting that CD34 is only expressed in 50% of cases, and the ES is characterized by the loss of SMARCB1/INI1 expression which is a tumor-suppressing gene localized at chromosome 22q [5,10]. Proximal-type ES constitutes a diagnostic difficulty due to the morphological and immunohistochemical similarities with other soft tissue tumors such as malignant rhabdoid tumors, epithelioid angiosarcoma, undifferentiated carcinoma, and other malignancies [14].

The recommended approach for treating localized cervical ES is complete resection with negative margins (R0) and no tumor rupture [1]. Adjuvant radiation therapy may be considered as part of the treatment plan [2]. Metastatic ES and locally advanced unresectable ES are associated with a poor prognosis [6]. The selection of therapy in palliative settings will be influenced by the patient's performance status, comorbidities, treatments received, and symptoms [1].

Many chemotherapeutic drugs and regimens have demonstrated activity in soft tissue sarcomas such as anthracycline-based regimens, gemcitabine-based regimens, and taxane [15]. ES is among the most chemoresistant sarcomas, the role of chemotherapy is not demonstrated, and the literature remains insufficient given the rarity of this tumor [6]. Tazemetostat is an oral targeted therapy anti-EZH2 (Enhancer of Zeste Homolog2 inhibitor) tested in a phase II basket trial including metastatic and advanced ESs with loss of SMARCB1/INI1 expression (the absence of its expression leads to overexpression of EZH2 by tumor cells) which demonstrated significant clinical benefits and it shows promise in improving the prognosis of this sarcoma [16]. A clinical trial is currently underway to evaluate the efficacy of the tazemetostat associated with doxorubicin as a frontline treatment for advanced ES [17]. Pazopanib a multi-kinase inhibitor was tested in phase III palette trials including patients with different non-adipocytic soft tissue sarcomas pretreated with chemotherapy that demonstrated a progression-free survival benefit without overall survival improvement [18]. Promising outcomes have been observed with immunotherapy tested in uncommon sarcomas including ES (phase II trial) [19]. It is necessary to develop new targets and effective therapies to improve the prognosis of this sarcoma which mostly relies on the disease's stage, location, rhabdoid differentiation, and responsiveness to therapy [2,5].

## Conclusions

ES is an aggressive and rare sarcoma with a worse prognosis. The clinical presentation differs depending on the location. The diagnosis was confirmed by anatomopathology and immunohistochemistry and interpreted carefully by an expert pathologist to rule out other potential diagnoses.

Providing treatment in palliative settings for this chemoresistant sarcoma can be rather challenging. Patients must be treated in specialized institutions or included in clinical trials. Developing novel and efficacious therapeutics is crucial for enhancing the prognosis of this sarcoma.
